# Long‐Term Course of Depression After Stroke and Risk Factors for Symptoms With Poor Progression: A Population‐Based Study

**DOI:** 10.1161/JAHA.125.041931

**Published:** 2025-07-29

**Authors:** Lu Liu, Iain J. Marshall, Ajay Bhalla, Xianqi Li, Salma Ayis, Charles D. A. Wolfe, Yanzhong Wang, Matthew D. L. O'Connell

**Affiliations:** ^1^ School of Life Course and Population Sciences King’s College London London United Kingdom; ^2^ NIHR Applied Research Collaboration (ARC) South London London United Kingdom; ^3^ Department of Ageing Health and Stroke Guy’s and St Thomas’ National Health Service Foundation Trust and King’s College London London United Kingdom

**Keywords:** depression, neurovascular disease, stroke, Cerebrovascular Disease/Stroke, Epidemiology, Mental Health, Risk Factors

## Abstract

**Background:**

Data on the long‐term poststroke depression trajectories and their determinants are limited. This study aims to estimate the 5‐year course of poststroke depression and identify risk factors for recurrent and persistent depression.

**Methods:**

Data were from the South London Stroke Register (1997–2022). Depression was defined as a subscale score >7 on the Hospital Anxiety and Depression Scale at 3 months and annually up to 5 years. Participants with >2 assessments of depression were included. Multinomial logistic regression examined associations between baseline factors, changes in function, and recurrent or persistent depression.

**Results:**

The analysis comprised 1724 participants (mean age, 65.5 years; men, 55.9%, White race, 65.2%). Of these, 1067 (61.9%) were not depressed at any time point. Among those with depression at some time point, 125 (19.0%) had transient depression, 231 (35.2%) had recurrent depression, and 301 (45.8%) had persistent depression. Patients with moderate to severe stroke (adjusted odds ratio, 1.81 [95% CI, 1.25–2.61]) or physical disability (adjusted odds ratio, 1.59 [95% CI, 1.12–2.26]) were more likely to develop recurrent depression, while patients with cognitive impairment (adjusted odds ratio, 2.09 [95% CI, 1.44–3.05]) or prestroke depression (adjusted odds ratio, 2.67 [95% CI, 1.60–4.47]) were at increased likelihood of having persistent depression. Patients exhibiting a decline in physical ability at 3 months were more likely to experience depression with poor progression (recurrent or persistent depression), independent of the initial severity of physical disability.

**Conclusions:**

Recurrent depression was associated with moderate to severe stroke or disability, whereas persistent depression was linked to prestroke depression or cognitive impairment. Progressive worsening disability was associated with recurrent or persistent depression, regardless of initial severity.

**Nonstandard Abbreviations and Acronyms**
AMT: Abbreviated Mental Test
BI: Barthel index
HADS: Hospital Anxiety and Depression Scale
HADS‐D: Hospital Anxiety and Depression Scale–Depression
NIHSS: National Institutes of Health Stroke Scale
PSD: poststroke depression
SLSR: South London Stroke Register

**Clinical Perspective**

**What Is New?**

- Patients with moderate to severe stroke or moderate to severe physical disability were more likely to develop recurrent depression, while cognitive impairment and prestroke depression were associated with persistent depression after stroke.
- Worsening physical disability was associated with unfavorable (recurrent or persistent) depression symptoms regardless of initial severity of physical disability.

**What Are the Clinical Implications?**

- Stroke survivors with recurrent depression may benefit from physical rehabilitation, while patients with persistent depression might respond more favorably to psychological therapy.

Depression is the most common mental health problem after stroke and is associated with poor functional outcomes and increased mortality rate in stroke survivors.^1^ Given the aging population and rising life expectancy in people living with stroke, addressing the challenge of poststroke depression (PSD) is becoming increasingly urgent. Understanding the natural history and course of PSD could help to inform prognosis. There are only a few studies estimating the course of depression beyond 1 year after stroke. According to a recent systematic review and meta‐analysis in 2023, only 5 studies reported the natural history of depression beyond 1 year after stroke and the results were inconclusive.^2^ Astrom et al demonstrated that 36% of patients had a long‐lasting depression up to 3 years after stroke,^3^ while Ayerbe et al found 6% were depressed at every assessment during a 5‐year follow‐up.^4^ Therefore, more research is needed to have a better understanding of the long‐term course of depression.

Stroke severity, cognitive impairment, physical disability, and prestroke depression are the recognized consistent risk factors for PSD.^5^, ^6^, ^7^, ^8^, ^9^, ^10^, ^11^ However, previous studies have estimated the associations of these factors and PSD on the basis of depression assessment at only 1 time point,^6^, ^7^, ^8^, ^9^, ^10^, ^11^ which are unable to capture how depressive symptoms change over time and may not provide a full picture between putative factors and the progression of PSD. Moreover, previous evidence largely focuses on the baseline predictors for PSD; there is a need for studies estimating the effect of changes in functional disabilities on the progression of PSD. Investigating these changing temporal patterns of depression and their associations, concurrently considering the role of changes in functional abilities is beneficial for identifying at‐risk individuals and implementing tailored prevention strategies. This will help arrange limited resources toward those most likely to follow patterns with recurrent or persistent depression.

To address this research gap, we used data from a population‐based study to (1) estimate the long‐term course of depression up to 5‐years after stroke, (2) identify stroke survivors' characteristics predictive of recurrent or persistent depression, and (3) assess the influence of changes in functional abilities on the progression of depression.

## Methods

### Availability of Data and Materials

Because of the sensitive nature of the data collected for this study, requests to access the data set for academic use should be made to the SLSR (South London Stroke Register) team (https://www.kcl.ac.uk/lsm/research/divisions/hscr/research/groups/stroke/index.aspx).

### Ethics Approval and Consent to Participate

The SLSR has ethical approval from the National Health Service Health Research Authority (Wales REC 1 Research Ethics Committee, reference number: 22/WA/0027), and previously from the ethics committees of Guy's and St Thomas' Hospital Trust, King's College Hospital, Queens Square, and Westminster Hospital (London, UK). All patients or their relatives gave written, informed consent to their participation in the study.

### Study Population

First‐ever stroke patients were recruited to the SLSR, an ongoing, prospective, population‐based cohort study covering a specific area in the inner city of London.^12^ Stroke was defined according to World Health Organization *International Classification of Diseases*, *Tenth Revision* (*ICD‐10*) criteria,^13^ with subtypes (ischemic and hemorrhagic stroke) defined by a study stroke physician, with reference to symptoms, neuroimaging, and other test results. The patients were registered during the acute phase (within 48 hours) of their first stroke. Data from patients registered in the SLSR between 1997 and 2017 and followed up to 2022 were used for this analysis.

### Baseline Data Collection

Data collected at the acute phase (within 7 days after stroke) included sociodemographic factors, physical disability, stroke severity, prestroke depression, and cognitive function. Demographic data included age, sex, race (White, Black, and other race), socioeconomic status, and stroke subtype (ischemic stroke or hemorrhagic stroke). The index of multiple deprivation was used to rank the socioeconomic status with higher score indicating more deprived. Data on prestroke depression were obtained from hospital or general practitioner records of diagnosis. Physical disability was assessed using the Barthel index (BI). The BI is numerical and scores 10 functions (feeding, grooming, bathing, dressing, bowel and bladder care, toilet use, ambulation, transfers, and stair climbing) on a scale from 0 to 20.^14^ A score of 0 to 14 was classified as moderate to severe disability, while a score of 15 to 20 was defined as mild disability.^15^ Stroke severity was measured using the National Institutes of Health Stroke Scale (NIHSS). The NIHSS score runs from 0 to 42 points, where the higher the points, the more severe stroke can be. A score on the NIHSS of 0 to 4 was used to identify patients with mild stroke, and a score of >5 was classified as moderate to severe stroke.^16^ Cognitive function was assessed with the Mini‐Mental State Examination before 2000 and with the Abbreviated Memory Test (AMT) after 2000. The Mini‐Mental State Examination consists of a variety of questions and has a maximum score of 30 points. The questions typically have been grouped into 7 categories, each rationally representing a different cognitive domain or function: orientation to time (5 points); orientation to place (5 points); registration of 3 words (3 points); attention and calculation (5 points); recall of 3 words (3 points); language (8 points), and visual construction (1 point).^17^ The AMT consists of 10 questions that evaluate orientation, memory, attention, and general knowledge; each correct answer scores 1 point, giving a maximum of 10 points. Example items from the AMT include questions such as “What is your age?” and “What is the current year?.”^18^ Those with a Mini‐Mental State Examination score of <24 (out of 30) or AMT score <8 (out of 10) were classified as having cognitive impairment.^17^, ^18^ Some patients were unable to undergo the cognitive test because of severe aphasia, dysphasia, or visual impairment.

### Follow‐Up

After initial assessment, patients underwent follow‐up interviews at 3 months, 1 year, then annually after stroke until March 2014. From March 2014 onward, patients were followed‐up at 3 months, 1 year, and 5 years after the initial assessment. Follow‐up data were collected by validated postal or face‐to‐face instruments with patients or their carers. Patients who were lost to follow‐up at 1 time point were captured at the following assessment. The Hospital Anxiety and Depression Scale (HADS) is a widely used questionnaire to assess depression and anxiety status. The HADS comprises 7 items each for depression and anxiety, with responses rated on a 0‐to‐3 scale. This results in 2 subscales, the HADS–Depression (HADS‐D) and the HADS–Anxiety, each yielding a score range of 0 to 21.^19^ At follow‐up, patients were screened for depression using the HADS–D subscale. HADS has been validated in patients with stroke and has shown a good performance both when it is used in a face‐to‐face interview and when it is self‐administered (Cronbach α >0.80; optimum performance when a HADS‐D subscale score >7 is used to identify depression; sensitivity, 0.73; specificity, 0.82).^20^ Despite its good performance, HADS is not a diagnostic scale but a screening tool that indicates risk of depression. However, we use the term *depression* in patients with scores >7 in this article for succinctness. The follow‐up interview included many other questionnaires in addition to the HADS. Usually, before the fieldworkers reached the HADS, they were able to judge the degree to which the patient may have cognitive impairment or aphasia. If it was evident that the patient was very confused, had difficulties in understanding the questions, or was unable to clearly express their thoughts, their responses would be invalid. Also, if the patient's speech was so poor that they were unable to effectively answer the HADS or are becoming distressed by the pressure of trying to speak when they were struggling to get the words out, the questions would not be asked, and the test would be terminated. Since depression cannot reliably be assessed by proxy, those with severe cognitive or communication problems that the fieldworker judged would give invalid responses were not assessed.

### Statistical Analysis

To capture the remitting–relapsing course of depression, we included patients with at least 3 assessments. Transient depression was defined as depression (ie, HADS‐D subscale score > 7) at only 1 time point. Persistent depression was defined as continuous presence of depression without recovery since the onset of the initial depression episode (ie, HADS‐D subscale score was not <8). Recurrent depression was defined as patients with depression who became depressed again after recovering from depression (ie, an interepisode HADS‐D score of <8, occurring between 2 episodes during which the HADS‐D subscale score was >7).

Baseline characteristics for individuals falling into the different depression trajectories (no depression, transient depression, recurrent depression, persistent depression) are presented as mean values, counts, and percentages where relevant. A χ^2^ test was used to compare the categorical covariates across different depression trajectories, and ANOVA was used to compare the continuous variables. Next, multinomial logistic regression models were used to identify predictors for PSD trajectory group memberships while considering the potential effects of other variables. Potential variables included age at baseline, socioeconomic status, sex (male and female), race (White, Black, and other), stroke subtype (ischemic and hemorrhagic stroke), stroke severity (mild and moderate to severe stroke), functional disability at baseline (mild and moderate to severe disability), cognition at baseline (normal cognitive function and cognitive impairment) and prestroke depression (yes or no). Change in functional ability between baseline and 3 months was classified into 4 categories: mild disability at baseline and 3 months, mild disability at baseline and moderate to severe at 3 months, moderate to severe disability at baseline and mild at 3 months, and moderate to severe disability at baseline and 3 months. Change in cognition was classified into 4 categories: normal cognition at baseline and 3 months, normal cognition at baseline and impaired at 3 months, impaired cognition at baseline and normal cognition at 3 months, and impaired cognition at both time points). For the logistic regression analyses, we fitted 2 models: model 1 was adjusted for age, sex, race, socioeconomic status, stroke subtype, stroke severity, physical disability, cognitive function, and prestroke depression; model 2 repeated using the change in physical disability and change in cognitive function instead of the baseline predictors. Variables with missing data of at least 10% had a separate category for missing data, for example, history of depression, 0 (no), 1 (yes), and 2 (missing). A further analysis was conducted to compare estimates obtained in multivariate analysis when the category for missing data was included and when it was not included. The results obtained using separate categories for missing data were presented in the main analysis.

Statistical significance was defined as a *P* value <0.05, and all analyses were conducted using 2‐tailed tests. All analyses we performed using Stata version 17.0 (StataCorp, College Station, TX).

### Sensitivity Analyses

To test the robustness of our study findings, we performed several sensitivity analyses. First, to assess whether continuous measures of key risk factors—physical disability (BI), stroke severity (NIHSS), and cognitive function (AMT)—predicted depression trajectories, we conducted a sensitivity analysis using these variables in their continuous form. Since the Mini‐Mental State Examination was only available before 2000 and only in categorical form, we used the AMT as a continuous measure of cognitive function for the purposes of this analysis. Furthermore, changes in functional disability were quantified continuously to determine whether incremental variations of 1 or 2 points on the BI predict subsequent depression trajectories. These analyses were stratified according to baseline disability status, distinguishing between participants with mild disability and those with moderate to severe disability. Second, treatment for depression may have an effect on the course of PSD. We conducted a further sensitivity analysis that excluded patients using antidepressants before stroke or at any time points after stroke. Third, patients with stroke have a high mortality rate. The main analyses were repeated in patients who had survived ≥5 years after stroke to assess the effect of death. Fourth, as the follow‐up frequency has changed since March 2014, patients recruited after March 2014 did not have depression assessed yearly. We conducted 1 sensitivity analysis, which included only patients registered before March 2014. Finally, the estimates were repeated in patients with complete follow‐up at the 6 time points.

## Results

### Sample Characteristics

Between 1997 and 2017, the SLSR registered 5756 patients, and 1263 patients died within 3 months after stroke. Figures S1–S12 provide details of patients recruited between 1997 and 2017. The proportions of patients who did not complete the HADS due to cognitive difficulties/aphasia at each time point (3 months, then annually up to 5 years) were 23.3% (628/2700), 16.5% (435/2638), 18.4% (312/1697), 16.0% (254/1587), 14.1% (193/1369), and 18.4% (352/1908), respectively. A total of 1724 patients who had ≥3 assessments were included in the analysis. The mean age of included patients was 65.5 ± 13.7 years, 55.9% were men, and White race comprised 65·2% and Black race comprised 27.2%. The proportion of patients who were unable to undergo the cognitive test was 28.8% (497/1724). A comparison of baseline characteristics between participants included in the final analysis and those excluded was shown in Tables S1–S12. Briefly, excluded patients were older and had more severe functional disabilities than those included.

### Long‐Term Course of Depression

A total of 1724 patients were included in estimating the 5‐year course of depression (mean±SD BI, 15.1±6.2; mean±SD NIHSS, 6.2±5.5; mean±SD AMT, 8.5±2.4. Among the included patients, 1067 (61.9%) were not depressed at any assessment, 657 patients experienced depression at ≥1 time points after stroke, with a cumulative incidence of 38.1% over the 5‐year follow‐up period. Of patients with depression at ≥1 time points, 125 (19.0%) had transient depression, 231 (35.2%) with depression recurred after recovery, and another 301 (45.8%) had persistent depression. Table 1 and Tables S2 and S3 summarizes the demographic characteristics, stroke severity, and functional disabilities of participants across the 4 groups. Compared with other individuals, patients with persistent depression were younger and had a higher proportion of cognitive impairment and prestroke depression. Individuals with recurrent depression had a higher proportion of moderate to severe stroke and moderate to severe physical disabilities.

**Table 1:** Baseline Characteristics of the Participants According to Depressive Symptom Categories

	No depression	Transient depression	Recurrent depression	Persistent depression
	(N=1067)	(N=125)	(N=231)	(N=301)
Age, y	66.0±13.8	64.8±13.1	65.9±12.7	63.6±14.1
Socioeconomic status (IMD score)*	33.7±10.5	32.8±11.1	36.5±9.8	36.1±9.2
Sex, n (%)				
Male	607 (56.9)	72 (57.6)	120 (52.0)	164 (54.5)
Female	400 (43.1)	53 (42.4)	111 (48.1)	137 (45.5)
Race, n (%)				
White	722 (67.7)	69 (55.2)	141 (61.0)	192 (63.8)
Black	276 (25.9)	46 (36.8)	73 (31.6)	74 (24.6)
Unknown/Others	69 (6.5)	10 (8.0)	17 (7.4)	35 (11.6)
Stroke, n (%)				
Ischemic stroke	907 (85.0)	100 (80.0)	196 (84.9)	258 (85.7)
Hemorrhagic stroke	153 (14.3)	24 (19.2)	34 (14.7)	42 (14.0)
Unknown	7 (0.7)	1 (0.8)	1 (0.4)	1 (0.3)
Stroke severity,† n (%)				
Mild stroke	444 (41.6)	44 (35.2)	66 (28.6	106 (35.2)
Moderate and severe stroke	345 (32.3)	52 (41.6)	112 (48.5)	124 (41.2)
Unknown	278 (26.1)	29 (23.2)	53 (22.9)	71 (23.6)
Physical disability,‡ n (%)				
Mild disability	647 (60.6)	60 (48.0)	104 (45.0)	158 (52.5)
Moderate to severe disability	281 (26.3)	44 (35.2)	89 (38.5)	102 (33.9)
Unknown	139 (13.0)	21 (16.8)	38 (16.5)	41 (13.6)
Change in physical disability, n (%)				
Mild at baseline and 3 mo	484 (45.4)	40 (32.0)	68 (29.4)	102 (33.9)
Mild at baseline and moderate to severe at 3 mo	8 (0.8)	2 (1.6)	15 (6.5)	11 (3.7)
Moderate to severe at baseline and mild at 3 mo	168 (15.8)	23 (18.4)	43 (18.6)	47 (15.6)
Moderate to severe at baseline and 3 mo	46 (4.3)	15 (12.0)	31 (13.4)	31 (10.3)
Unknown	361 (33.8)	45 (36.0)	74 (32.0)	110 (36.5)
Cognitive function,§ n (%)				
Normal cognition	635 (59.5)	61 (48.8)	122 (52.8)	148 (49.2)
Cognitive impairment	127 (11.9)	26 (20.8)	43 (18.6)	65 (21.6)
Unknown	305 (28.6)	38 (30.4)	66 (28.6)	88 (29.2)
Change in cognitive function, n (%)				
Normal at baseline and 3 mo	444 (41.6)	40 (32.0)	89 (38.5)	87 (28.9)
Normal at baseline and impaired at 3 mo	32 (3.0)	8 (6.4)	11 (4.8)	14 (4.7)
Impaired at baseline and normal at 3 mo	56 (5.3)	7 (5.6)	23 (10.0)	23 (7.6)
Impaired at baseline and 3 mo	35 (3.3)	10 (8.0)	12 (5.2)	26 (8.6)
Unknown	500 (46.9)	60 (48.0)	96 (41.6)	151 (50.2)
Prestroke depression, n (%)				
No	811 (76.0)	92 (73.6)	169 (73.2)	225 (74.8)
Yes	43 (4.0)	8 (6.4)	16 (6.9)	30 (10.0)
Unknown	213 (20.0)	25 (20.0)	46 (19.9)	46 (15.3)

The χ^2^ test was used to compare the categorical covariates across different depression trajectories, and ANOVA was used to compare the continuous variables. IMD indicates index of multiple deprivation.

* Higher scores on the IMD indicate greater deprivation.

^†^ Moderate to severe stroke: National Institutes of Health Stroke Scale score >4.

^‡^ Moderate to severe physical disability: Barthel index<15.

^§^ Cognitive impairment: Mini‐Mental State Examination <24 or Abbreviated Mental Test <8.

### Predictors for Different Patterns of Depression

The adjusted odd ratios (aORs) and 95% CIs for the association between patients' characteristics and the 4 depression patterns (model 1) are presented in Table 2. Compared with patients with no depression throughout the follow‐up, the more deprived were more likely to have recurrent (aOR, 1.02 [95% CI, 1.01–1.04]) or persistent depression (aOR, 1.02 [95% CI, 1.01–1.03]). Patients with moderate to severe stroke (aOR, 1.81 [95% CI, 1.25–2.61]) or moderate to severe physical disabilities (aOR, 1.59 [95% CI, 1.12–2.26]) were at increased likelihood of being in the recurrent depression group, while patients with cognitive impairment (aOR, 2.09 [95% CI, 1.44–3.05]) or prestroke depression (aOR, 2.67 [95% CI, 1.60–4.47]) were significantly more likely to have persistent depression. Patients with older age were less likely to be in the group of persistent depression (aOR, 0.98 [95% CI, 0·97–0.99]). Results using patients with complete data for all covariates were similar to the results using separate categories for missing data (Table S4).

**Table 2:** Associations Between Patients' Baseline Characteristics and Courses of Depressive Symptoms Up to 5 Years After Stroke

Transient depression	Recurrent depression			Persistent depression		
	aOR (95%CI)	*P* value	aOR (95%CI)	*P* value	aOR (95%CI)	*P* value
Age	1.00 (0.98–1.01)	0.65	1.00 (0.99–1.01)	0.97	0.98 (0.97–0.99)	<0.01
Socioeconomic status (IMD score) *	0.99 (0.97–1.00)	0.15	1.02 (1.01–1.04)	<0.01	1.02 (1.01–1.03)	<0.01
Sex						
Male	Reference					
Female	1.02 (0.69–1.52)	0.91	1.17 (0.99–1.01)	0.30	1.11 (0.85–1.46)	0.45
Race						
White	Reference					
Black	1.81 (1.18–2.80)	<0.01	1.24 (0.88–1.73)	0.22	0.84 (0.61–1.16)	0.28
Stroke subtype						
Ischemic stroke						
Hemorrhagic stroke	1.19 (0.70–2.03)	0.53	0.99 (0.64–1.52)	0.96	0.86 (0.58–1.28)	0.45
Stroke severity†						
Mild stroke	Reference					
Moderate and severe stroke	1.18 (0.73–1.91)	0.50	1.81 (1.25–2.61)	<0.01	1.21 (0.87–1.69)	0.27
Physical disability‡						
Mild disability	Referenced					
Moderate to severe disability	1.34 (0.83–2.15)	0.23	1.59 (1.12–2.26)	0.01	1.30 (0.94–1.79)	0.12
Cognitive function§						
Normal cognition	Reference					
Cognitive impairment	1.61 (0.92–2.82)	0.10	1.35 (0.88–2.05)	0.17	2.09 (1.44–3.05)	<0.01
Prestroke depression						
No	Reference					
Yes	2.19 (0.97–1.00)	0.15	1.88 (1.00–3.56)	0.05	2.67 (1.60–4.47)	<0.01

Multinomial logistic regression was used to identify factors associated with distinct trajectory groups. Group of no depression was the reference group. aOR indicates adjusted odds ratio; and IMD, index of multiple deprivation.

* Higher scores on the IMD indicate greater deprivation.

^†^ Moderate to severe stroke: National Institutes of Health Stroke Scale score >4.

^‡^ Moderate to severe physical disability: Barthel index <15.

^§^ Cognitive impairment: Mini‐Mental State Examination <24 or Abbreviated Mental Test <8.

Model 2 estimated the associations between change in cognitive function/change in physical disability and 4 trajectory groups. Compared with patients with normal cognition at baseline and 3 months, those with impaired cognition at baseline and normal at 3 months (aOR, 1.96 [95% CI, 1.11–3.45]) or those with impaired cognition at baseline and 3 months (aOR 3.51, 95% CI 1.92–6.41) were more likely to have persistent depression, while those with normal cognition at baseline and impaired cognition at 3 months had similar risks of developing persistent depression. Individuals with mild physical disability at baseline and moderate to severe disability at 3 months were more likely to develop both recurrent (aOR, 11.60 [95% CI, 4.56–29.68]) and persistent depression (aOR, 5.59 [95% CI, 2.08–15.03]) when comparing with patients with mild disability at baseline and 3 months. Similarly, individuals with moderate to severe disability at baseline and 3 months were at increased risks of having depression with poor progression (recurrent depression: aOR, 3.97 [95% CI, 2.24–7.02]; persistent depression: aOR, 2.29 [95% CI, 1.31–4.02]). However, the likelihood for being in persistent depression group was not significantly different for individuals with moderate to severe disability at baseline and mild disability at 3 months when compared with the reference group (aOR, 1.12 [95% CI, 0.79–1.84]). Results are shown in Table 3.

**Table 3:** Associations Between Changes in Patients' Characteristics and Courses of Depressive Symptoms Up to 5 Years After Stroke

Transient depression	Recurrent depression			Persistent depression		
	aOR (95%CI)	*P* value	aOR (95%CI)	*P* value	aOR (95%CI)	*P* value
Age	0.99 (0.98–1.01)	0.47	1.00 (0.98–1.01)	0.53	0.98 (0.97–0.99)	<0.01
Socioeconomic status (IMD score) *	0.99 (0.97–1.00)	0.13	1.02 (1.01–1.04)	<0.01	1.02 (1.00–1.03)	<0.01
Sex						
Male	Reference					
Female	0.98 (0.66–1.46)	0.92	1.08 (0.79–1.46)	0.64	1.06 (0.81–1.40)	0.67
Race						
White	Reference					
Black	1.76 (1.14–2.74)	0.01	1.30 (0.92–1.83)	0.14	0.83 (0.60–1.16)	0.27
Stroke subtype						
Ischemic stroke						
Hemorrhagic stroke	1.23 (0.72–2.10)	0.46	1.03 (0.67–1.59)	0.88	0.91 (0.61–1.35)	0.63
Stroke severity†						
Mild stroke	Reference					
Moderate and severe stroke	1.17 (0.73–1.87)	0.52	1.74 (1.21–2.51)	<0.01	1.22 (0.88–1.69)	0.24
Change in physical disability‡						
Mild at baseline and 3 mo	Reference					
Mild at baseline and moderate to severe at 3 mo	2.67 (0.52–13.63)	0.24	11.60 (4.56–29.68)	<0.01	5.59 (2.08–15.03)	<0.01
Moderate to severe at baseline and mild at 3 mo	1.56 (0.87–2.81)	0.14	1.61 (1.03–2.51)	0.04	1.21 (0.79–1.84)	0.38
Moderate to severe at baseline and 3 mo	3.03 (1.39–6.61)	<0.01	3.97 (2.24–7.02)	<0.01	2.29 (1.31–4.02)	<0.01
Change in cognitive function§						
Normal at baseline and 3 mo	Reference					
Normal at baseline and impaired at 3 mo	2.34 (0.97–5.60)	0.06	1.00 (0.46–2.18)	0.99	1.70 (0.82–3.49)	0.15
Impaired at baseline and normal at 3 mo	0.56 (0.17–1.90)	0.35	1.51 (0.85–2.68)	0.16	1.96 (1.11–3.45)	0.02
Impaired at baseline and 3 mo	2.12 (0.91–4.96)	0.08	1.06 (0.50–2.23)	0.88	3.51 (1.92–6.41)	<0.01
Prestroke depression						
No	Reference					
Yes	2.30 (1.02–5.18)	0.04	2.05 (1.08–3.91)	0.03	2.83 (1.69–4.76)	<0.01

Multinomial logistic regression was used to identify factors associated with distinct trajectory groups. Group of no depression was the reference group. aOR indicates adjusted odds ratio; and IMD, index of multiple deprivation.

* Higher scores on the IMD indicate greater deprivation.

^†^ Moderate to severe stroke: National Institutes of Health Stroke Scale score > 4.

^‡^ Moderate to severe physical disability: Barthel index<15.

^§^ Cognitive impairment: Mini‐Mental State Examination <24 or Abbreviated Mental Test <8.

### Sensitivity Analyses

First, findings from the sensitivity analysis, in which scale scores were treated as continuous variables, were similar to those of the primary analysis (Table S5). Among participants with mild disability at baseline, improvements of 1 or 2 points on the BI were not associated with a reduced risk of adverse depressive outcomes. However, a decline of just 1 point was linked to a significantly increased likelihood of recurrent or persistent depression. In contrast, within the cohort showing moderate to severe baseline disability, gains in BI scores did not confer a differential risk of unfavorable depression trajectories compared with stable scores. Results are shown in Table S6. The subgroup of patients with moderate to severe disability who experienced a worsening in BI (n=31) was too small to support a reliable logistic regression, so the model estimating the effect of BI decline on distinct trajectories in this stratum was not conducted. Second, in the analysis excluding patients taking antidepressants at any time point, a total of 120 patients were excluded. Among the 1604 patients who did not take antidepressants before stroke or during the 5‐year follow‐up, 1024 (63.8%) were not depressed at any time point. Among patients with depression at some point (n=580), 113 (19.5%) had transient depression, 207 (35.7%) had recurrent depression and 260 (44.8%) had persistent depression. The risk factors associated in the sensitivity analysis were similar to those in the main analysis except that prestroke depression was not associated with recurrent depression or persistent depression (Tables S7 and S8). Third, among the 1561 patients surviving >5 years after stroke, 948 were not depressed at any time points. Of the 613 who had depression at ≥1 assessments, 118 (19.3%) had transient depression, 222 (36.3%) had recurrent depression, and 273 (44.5%) had persistent depression. Fourth, 1599 patients were recruited before 2014, and 986 patients were not depressed at any assessments. Among the 613 patients with depression at >1 time point, 118 (19.3%) had transient depression, 222 (36.3%) had recurrent depression, and 273 (44.5%) had persistent depression. The risk factors for recurrent depression and persistent depression in these 2 sensitivity analyses (the third and fourth ones) were consistent with that in the main analysis (Tables S9–S12). Among the patients who were assessed at all 6 time points (n=276), 157 (57.5%) were not depressed at any assessment. Of the patients who had depression at ≥1 time points, 21 (18.6%) had transient depression, 59 (48.8%) were depressed at every assessment, and another 36 (31.0%) had recurrent depression. The risk factor analysis (multinomial logistic regression) was not performed in this sensitivity analysis due to the small sample size.

## Discussion

In the present study, we found that depression affects about 40% (657/1724) of stroke survivors during the 5‐year follow‐up. Among patients with depression at ≥1 time points, about 80% had poor progression (recurrent or persistent depression) and only 20% had transient depression with no recurrence. Patients with moderate to severe stroke or moderate to severe physical disabilities were more likely to have recurrent depression, whereas cognitive impairment and prestroke depression were associated with the development of persistent depression. Worsening physical disabilities during the first 3 months after stroke were related to poor progression irrespective of the baseline physical ability.

Our earlier analysis on the natural history of depression with follow‐up for up to 18 years after stroke found that 90% of incident depression occurred within 5 years after stroke.^21^ As a result, we focused on analyzing the developmental patterns of depression over a period of 5 years. Several longitudinal studies have estimated the long‐term course of depression.^3^, ^22^ Robinson et al found that of patients who were depressed in the hospital, 37% had no symptoms of depression, and the remaining 63% continued to have depression at 2 years after stroke.^22^ Astrom et al demonstrated that 36% patients had a long‐lasting depression up to 3 years after stroke.^3^ In the current study, we found that 45% of patients with depression developed persistent depression, about 35% experienced depression recurrence, and only about 20% had transient depression. The inconsistent results may be due to heterogeneity in sample sizes and methods of assessing depression. Another explanation is the differences in follow‐up frequency and follow‐up period. As PSD has a short duration within 1 year, a long duration between 2 follow‐up times may miss the recovery period, leading to misclassification of recurrent depression to persistent depression. Our findings imply that once patients develop depression, ongoing monitoring and close follow‐up should be implemented as they are at a very high risk of having a poor prognosis (recurrent depression or persistent depression).

There is a general consensus that severe stroke, cognitive impairment, physical disability, and prestroke depression are consistently associated with the development of PSD at 1 time point.^1^ However, how these factors affect the course and progression in patients with PSD has not been evaluated before. In the present study, we found that compared with patients with no depression, patients with history of depression or baseline cognitive impairment were more likely to have recurrent depression, while moderate to severe stroke and moderate to severe physical disability were associated with the development of persistent depression. One possible reason for this could be that history of depression has the largest impact on the development of PSD, and persistent depression is more serious than remitting–relapsing depression.^23^, ^24^ The other attribution may be that cognition and mood are linked above physical independence.^24^ The association between stroke severity or physical disability and the development of recurrent depression may be explained by the mechanism of depression recurrence. Even though the exact cause of recurrent depression is not fully understood, the reigning theory has been stress sensitization.^25^ As a consequence of major stressors (traumatic events) triggering early depressive episodes, individuals become increasingly vulnerable to recurrences, then lower levels of life stress triggered subsequent recurrence.^25^ Sudden stroke is a traumatic event for the sufferers, which triggers incident depression. When stroke survivors partially recover from stroke during rehabilitation, depressive symptoms may remit. However, it is difficult for patients with severe stroke to fully recover to normal functional ability and when functional recovery reaches a plateau, relapsing symptoms may be triggered by these less severe life stress (mild or moderate physical disabilities). We should notice that the *P* value for the observed association is borderline; further studies with larger sample size are needed to confirm or rebut our results. In the sensitivity analysis excluding patients who were taking antidepressant medication, prestroke depression was not significantly associated with either recurrent or persistent depression. This finding is likely attributable to the exclusion of a subset of individuals with prestroke depression who were undergoing antidepressant treatment. Further research, particularly randomized controlled trials, is needed to elucidate the impact of antidepressant treatment on the longitudinal course of poststroke depression.

Our findings on the association between putative risk factors and different patterns of depression symptom could facilitate early identification of unfavorable progression of PSD so that tailored prevention strategies could be implemented to improve outcomes and recovery. These findings may also have important therapeutic implications: stroke survivors with recurrent depression may be more likely to benefit from physical therapy for activities of daily living, while patients with persistent depression symptoms might be more likely to benefit from psychological therapy.

Our finding of the protective effect of older age in persistent depression symptoms is consistent with some of the previous literature. A meta‐analysis of 36 studies on PSD found that age >70 years nearly halved the risk of depression.^24^ It is unclear whether the age differences result from a biological change in aging, different psychosocial processes, or underdiagnosis of older adult patients. One possible explanation is that it is more difficult for young patients to adapt to the sudden onset of medical impairments, while older patients are better prepared to handle the stroke‐induced changes.^7^

In the present study, we found that the more deprived were more likely to have unfavorable depression symptoms. Regarding the association of socioeconomic status and PSD, there are still major discrepancies in the literature.^26^ These differences may be due to heterogeneity in sample sizes, follow‐up times, and methods of assessing depression.

Whereas multiple studies showed an association between poor functional outcome and PSD, fewer studies evaluated changes in functional disabilities in relation to the dynamic courses of depression after stroke. In the present study, we found that patients with worsening physical disabilities at 3 months after stroke were more likely to have unfavorable depression symptoms (recurrent and persistent depression), while those with moderate to severe disability at baseline and improvement at 3 months were not at increased risk of having persistent depressive symptoms. In other words, improvement in physical ability was protective for developing unfavorable depressive symptoms regardless of the initial severity of physical disability. In contrast with findings for physical disability, only baseline and not change in cognitive function was associated with persistent depression. There are discrepancies in the literature in relation to the association between functional decline and depression. In a multicenter prospective cohort study assessing the association of depression status at 3 months and 1 year and functional decline, Husseini et al concluded that no worsening or improvement in functional status might lead to improved mood.^27^ The other study found the absolute disability scores was a stronger determinant of depression than change in disability scores between 2 time points.^28^ There findings suggest that improving functional ability within 3 months is more important than the absolute functional status in prevention of depression. Clinicians are recommended to encourage patients to participate in rehabilitation actively, especially for patients with moderate to severe disabilities since improvement in BI could reduce the risks of developing PSD. However, these findings should be read with caution, as the CIs in the comparison between patients with mild baseline disability and moderate to severe disability at 3 months and the reference group is wide. This may partially be due to patients with mild or no disability being unlikely to have worsening disabilities. Therefore, we could not rule out the possibility that the estimate of the observed association in these patients may be less accurate due to small sample size.

### Strengths and Limitations

The general strengths of our study include its prospective design, population‐based sample, repeated assessment of depressive symptoms at multiple time points, and detailed adjustment for confounding. This study also has several limitations. First, the analyses were restricted to patients with at least 3 assessments of depression. The included patients had less severe strokes than the general stroke survivors. Therefore, the present study was more likely to include patients with mild and moderate stroke, limiting generalizability of our results to all stroke survivors. Second, as in almost all cohort studies, there are missing data in the follow‐ups. This was partially because of the difficulty in following up patients for such long time as well as the high mortality rate in patients with stroke. Even though the identified trajectories in patients with complete follow‐up at all time‐points were similar to those in the main analysis, selection bias may still be present as some characteristics of patients not responding to the HADS differed from patients included in the analysis. Third, depression is not diagnosed with a clinical interview, as it would have been unfeasible to assess with the *Diagnostic and Statistical Manual of Mental Disorders*, *Fourth Edition*, so many patients for such a long period of time. Although HADS is a recognized screening tool for PSD, the gold standard for diagnosis remains through clinician‐administered structured clinical interviews.^29^ Another limitation is that in the model assessing the association between changes in functional disability and different PSD patterns, the sample size in some category is not large enough to build a stable logistic model.

## Conclusions

In conclusion, patients with severe stroke or severe physical disability were more likely to develop recurrent depression, while cognitive impairment and prestroke depression were associated with persistent depression. Worsening physical disability was associated with poor progression regardless of initial severity of physical disability.

## Sources of Funding

This project is funded by the National Institute for Health and Care Research Programme under its Programme Grants for Applied Research (NIHR202339) and is supported by the National Institute for Health and Care Research Programme Applied Research Collaboration South London at King's College London National Health Service Foundation. L.L. received financial support from the China Scholarship Council PhD Scholarship (CSC No. 202108310074). The funders had no role in the study design, data collection and analysis, decision to publish, or preparation of the manuscript.

## Disclosures

None.

## Supporting information

Tables S1–S12
Figure S1
