# Supplementary material for: Long‐Term Course of Depression After Stroke and Risk Factors for Symptoms With Poor Progression: A Population‐Based Study
Source: J Am Heart Assoc. 2025 Jul 29;14(15):e041931. doi: 10.1161/JAHA.125.041931 (PMC12449949; doi:10.1161/JAHA.125.041931)
Supplement: Supplementary file 1 — Tables S1–S12 Figure S1 [file JAH3-14-e041931-s001.pdf]

Supplemental Materials

Table S1 Comparison of baseline characteristics between participants included (n = 1724) and excluded due to less than three assessments of depression (n = 2763)

	Included (N=1724)	Excluded (N=2763)	P value	Statistic result	Degree of freedom
**Age**	65.5±13.7	69.0±15.7	<0.01	-7.7	4485
**Socioeconomic status (IMD score)***	34.5±10.3	34.8±9.7	0.35	-0.9	4240
**Sex**			0.04	4.2	1
Male	963(55.9)	1457(52.7)			
Female	761(44.1)	1306(47.3)			
**Ethnicity**			<0.01	13.6	2
White	1124(65.2)	1657(60.0)			
Black	469(27.2)	837(30.3)			
Unknown/Others	131(7.6)	269(9.7)			
**Stroke**			0.07	5.2	2
Ischemic stroke	1461(84.7)	2338(84.7)			
Haemorrhagic stroke	253(14.7)	390(14.1)			
Unknown	10(0.6)	35(1.3)			
**Stroke severity†**			<0.01	26.9	2
Mild stroke	660(38.3)	860(31.1)			
Moderate to severe stroke	633(36.7)	1073(38.8)			
Unknown	431(25.0)	830(30.0)			
**Physical disability‡**			<0.01	77.9	2
Mild disability	969(56.2)	1214(44.0)			
Moderate to severe disability	516(29.9)	1176(42.5)			
Unknown	239(13.9)	373(13.5)			
**Cognitive function§**			<0.01	185.8	2
Normal cognition	966(56.0)	979(35.4)			
Cognitive impairment	261(15.1)	556(20.1)			
Unknown	497(28.8)	1228(44.4)			
**Pre-stroke depression**			<0.01	17.4	2
No	1307(75.8)	2131(77.1)			
Yes	87(5.1)	239(8.7)			
Unknown	330(19.1)	393(14.2)			

Note: Chi-squared test was used to compare the categorical covariates across different depression trajectories and ANOVA was used to compare the continuous variables.

*: Index of multiple deprivation, higher scores on the IMD indicate greater deprivation

†: Moderate to severe stroke: NIHSS score>4

‡: Moderate to severe physical disability: Barthel Index<15

§: Cognitive impairment: MMSE<24 or AMT<8

Table S2. Mean scores and range of Barthel Index, NIHSS score and AMT score.

	Barthel Index		NIHSS		AMT	
	**Mean (SD)**	**Range**	**Mean (SD)**	**Range**	**Mean (SD)**	**Range**
No depression	15.9(5.9)	(0,20)	5.5(5.0)	(0,31)	8.7(2.2)	(0,10)
Transient depression	13.9(6.5)	(0,20)	6.9(5.6)	(0,31)	8.1(2.7)	(0,10)
Recurrent depression	13.5(6.5)	(0,20)	7.5(6.0)	(0,31)	8.2(2.6)	(0,10)
Persistent depression	14.1(6.7)	(0,20)	7.2(6.1)	(0,31)	7.9(2.8)	(0,10)

Table S3 Baseline characteristics of the participants according to depression categories

	No depression (N=1067)	Transient depression (N=125)	Recurrent depression (N=231)	Persistent depression (N=301)	P value	Statistic result	Degree of freedom
**Age**	66.0±13.8	64.8±13.1	65.9±12.7	63.6±14.1	0.05	2.68	1720
**Socioeconomic status (IMD score)** *	33.7±10.5	32.8±11.1	36.5±9.8	36.1±9.2	<0.01	8.37	1632
**Sex**					0.52	2.2757	3
Male	607(56.9%)	72(57.6%)	120(52.0%)	164(54.5%)			
Female	400(43.1%)	53(42.4%)	111(48.1%)	137(45.5%)			
**Ethnicity**					<0.01	19.226	6
White	722(67.7%)	69(55.2%)	141(61.0%)	192(63.8%)			
Black	276(25.9%)	46(36.8%)	73(31.6%)	74(24.6%)			
Unknown/Others	69(6.5%)	10(8.0%)	17(7.4%)	35(11.6%)			
**Stroke**					0.82	2.928	6
Ischemic stroke	907(85.0%)	100(80.0%)	196(84.9%)	258(85.7%)			
Haemorrhagic stroke	153(14.3%)	24(19.2%)	34(14.7%)	42(14.0%)			
Unknown	7(0.7%)	1(0.8%)	1(0.4%)	1(0.3%)			
**Stroke severity†**					<0.01	27.849	6
Mild stroke	444(41.6%)	44(35.2%)	66(28.6%)	106(35.2%)			
Moderate and severe stroke	345(32.3%)	52(41.6%)	112(48.5%)	124(41.2%)			
Unknown	278(26.1%)	29(23.2%)	53(22.9%)	71(23.6%)			
**Physical disability‡**					<0.01	26.595	6
Mild disability	647(60.6%)	60(48.0%)	104(45.0%)	158(52.5%)			
Moderate to severe disability	281(26.3%)	44(35.2%)	89(38.5%)	102(33.9%)			
Unknown	139(13.0%)	21(16.8%)	38(16.5%)	41(13.6%)			
**Change in physical disability**					<0.01	88.505	12
Mild at baseline and 3m	484(45.4%)	40(32.0%)	68(29.4%)	102(33.9%)			
Mild at baseline and moderate to severe at 3m	8(0.8%)	2(1.6%)	15(6.5%)	11(3.7%)			
Moderate to severe at baseline and mild at 3m	168(15.8%)	23(18.4%)	43(18.6%)	47(15.6%)			
Moderate to severe at baseline and 3m	46(4.3%)	15(12.0%)	31(13.4%)	31(10.3%)			
Unknown	361(33.8%)	45(36.0%)	74(32.0%)	110(36.5%)			
**Cognitive function§**					<0.01	26.740	6
Normal cognition	635(59.5%)	61(48.8%)	122(52.8%)	148(49.2%)			

Cognitive impairment	127(11.9%)	26(20.8%)	43(18.6%)	65(21.6%)			
Unknown	305(28.6%)	38(30.4%)	66(28.6%)	88(29.2%)			
**Change in cognitive function**					<0.01	44.384	12
Normal at baseline and 3m	444(41.6%)	40(32.0%)	89(38.5%)	87(28.9%)			
Normal at baseline and impaired at 3m	32(3.0%)	8(6.4%)	11(4.8%)	14(4.7%)			
Impaired at baseline and normal at 3m	56(5.3%)	7(5.6%)	23(10.0%)	23(7.6%)			
Impaired at baseline and 3m	35(3.3%)	10(8.0%)	12(5.2%)	26(8.6%)			
Unknown	500(46.9%)	60(48.0%)	96(41.6%)	151(50.2%)			
**Pre-stroke depression**					<0.01	18.846	6
No	811(76.0%)	92(73.6%)	169(73.2%)	225(74.8%)			
Yes	43(4.0%)	8(6.4%)	16(6.9%)	30(10.0%)			
Unknown	213(20.0%)	25(20.0%)	46(19.9%)	46(15.3%)			

Note: Chi-squared test was used to compare the categorical covariates across different depression trajectories and ANOVA was used to compare the continuous variables.

*: Index of multiple deprivation, higher scores on the IMD indicate greater deprivation

†: Moderate to severe stroke: NIHSS score>4.

‡: Moderate to severe physical disability: Barthel Index<15.

§: Cognitive impairment: MMSE<24 or AMT<8.

Table S4 Associations between patient' baseline characteristics and courses of depression up to 5-years after stroke (sensitivity analysis, complete data analysis)

	Transient depression		Recurrent depression		Persistent depression	
	aOR(95%CI)	P value	aOR(95%CI)	P value	aOR(95%CI)	P value
**Age**	1.01(0.98-1.03)	0.66	1.00(0.98-1.02)	0.95	0.98(0.97-1.00)	0.04
**Socioeconomic status (IMD score)***	0.99(0.96-1.02)	0.49	1.03(1.01-1.05)	0.02	1.02(1.00-1.04)	0.02
**Sex**						
Male			Ref			
Female	0.90(0.49-1.64)	0.73	1.22(0.78-1.90)	0.39	1.07(0.73-1.59)	0.72
**Ethnicity**						
White			Ref			
Black	1.41(0.75-2.64)	0.29	1.19(0.74-1.93)	0.47	0.69(0.44-1.08)	0.10
**Stroke subtype**						
Ischemic stroke						
Haemorrhagic stroke	0.92(0.36-2.35)	0.87	0.54(0.24-1.21)	0.14	1.30(0.76-2.22)	0.35
**Stroke severity†**						
Mild stroke			Ref			
Moderate and severe stroke	1.54(0.80-2.97)	0.19	1.66(1.04-2.64)	0.03	1.53(0.99-2.36)	0.06
**Physical disability‡**						
Mild disability						
Moderate to severe disability	1.37(0.70-2.67)	0.35	1.69(1.03-2.77)	0.04	1.04(0.66-1.63)	0.88
**Cognitive function§**						
Normal cognition			Ref			
Cognitive impairment	1.26(0.62-2.55)	0.53	1.32(0.77-2.26)	0.31	2.00(1.27-3.15)	<0.01
**Pre-stroke depression**						
No			Ref			
Yes	1.53(0.43-5.40)	0.51	1.58(0.64-3.90)	0.32	2.42(1.18-4.97)	0.02

Note: Multinomial logistic regression was used to identify factors associated with distinct trajectory groups. Group of no depression was the reference group.

a: Index of multiple deprivation, higher scores on the IMD indicate greater deprivation

b: Moderate to severe stroke: NIHSS score>4

c: Moderate to severe physical disability: Barthel Index<15

d: Cognitive impairment: MMSE<24 or AMT<8

Table S5 Associations between patients' baseline characteristics and courses of depressive symptoms up to 5-years after stroke (Barthel Index score, NIHSS score and AMT score were treated as continuous form)

	Incident depression		Recurrent depression		Persistent depression	
	**aOR(95%CI)**	**P value**	**aOR(95%CI)**	**P value**	**aOR(95%CI)**	**P value**
**Age**	1.00(0.98-1.02)	0.85	1.00(0.98-1.01)	0.81	0.98(0.97-0.99)	<0.01
**Socioeconomic status (IMD score)***	0.98(0.96-1.00)	0.10	1.02(1.00-1.04)	0.03	1.02(1.00-1.04)	0.03
**Sex**						
Male			Ref			
Female	0.92(0.53-1.61)	0.78	1.22(0.81-1.83)	0.35	1.12(0.77-1.62)	0.56
**Ethnicity**						
White			Ref			
Black	1.60(0.89-2.88)	0.11	1.21(0.77-1.89)	0.41	0.69(0.45-1.06)	0.09
**Stroke subtype**						
Ischemic stroke			Ref			
Haemorrhagic stroke	1.00(0.43-2.30)	0.99	0.72(0.37-1.41)	0.34	1.32(0.79-2.21)	0.29
**Stroke severity†**	1.01(0.94-1.09)	0.70	1.81(1.25-2.61)	<0.01	1.05(1.01-1.10)	0.02
**Physical disability‡**	0.96(0.91-1.02)	0.20	0.95(0.91-0.99)	0.03	1.00(0.96-1.04)	0.99
**Cognitive function§**	0.96(0.85-1.08)	0.51	0.95(0.87-1.03)	0.23	0.91(0.84-0.98)	0.01
**Pre-stroke depression**						
No			Ref			
Yes	1.62(0.46-5.73)	0.45	1.66(0.68-4.10)	0.27	2.54(1.24-5.21)	0.01

Note: Multinomial logistic regression was used to identify factors associated with distinct trajectory groups. Group of no depression was the reference group.

*: Index of multiple deprivation, higher scores on the IMD indicate greater deprivation.

†: Stroke severity was measured by NIHSS score, higher scores indicate more severe stroke

‡: Physical disability was measured by Barthel Index, higher scores indicate less severe disability.

§: Cognitive impairment was measured by AMT.

Table S6 Associations between changes in Barthel Index score (treated as continuous variable) and courses of depressive symptoms up to 5-years after stroke

	Incident depression		Recurrent depression		Persistent depression	
	**aOR(95%CI)**	**P value**	**aOR(95%CI)**	**P value**	**aOR(95%CI)**	**P value**
**Mild disability at baseline**						
**1 point improvement in BI score at 3m**	0.81(0.49-1.33)	0.41	1.11(0.78-1.56)	0.57	0.99(0.68-1.45)	0.98
**2 points improvements in BI score at 3m**	0.44(0.11-1.82)	0.52	0.85(0.41-1.73)	0.65	0.93(0.42-2.08)	0.86
**1 point decrease in BI score at 3m**	1.43(1.18-1.74)	<0.01	1.53(1.33-1.76)	<0.01	1.37(1.19-1.57)	<0.01
**Severe disability at baseline**						
**1 point improvement in BI score at 3m**	0.99(0.9-1.08)	0.82	0.94(0.87-1.00)	0.07	0.74(0.15-3.77)	0.72
**2 points improvements in BI score at 3m**	1.02(0.92-1.13)	0.69	0.95(0.88-1.02)	0.17	0.96(0.89-1.04)	0.30

Note: Multinomial logistic regression was used to examine the effect of changes in physical disability on distinct trajectory groups. Group of no depression was the reference group. The subgroup of patients with moderate to severe disability who experienced a worsening in BI (n = 31) was too small to support a reliable logistic regression, so the model estimating the effect of BI decline on distinct trajectories in this stratum was not conducted.

Table S7: Associations between patients' baseline characteristics and courses of depressive symptoms up to 5-years after stroke (sensitivity analysis, exclude patients receiving antidepressant treatment)

	Incident depression		Recurrent depression		Persistent depression	
	aOR(95%CI)	P value	aOR(95%CI)	P value	aOR(95%CI)	P value
**Age**	1.00(0.98-1.01)	0.69	1.00(0.99-1.02)	0.63	0.98(0.97-0.99)	<0.01
**Socioeconomic status (IMD score)***	0.99(0.97-1.01)	0.21	1.02(1.01-1.04)	0.01	1.02(1.01-1.04)	<0.01
**Sex**						
Male			Ref			
Female	1.08(0.71-1.63)	0.73	1.18(0.87-1.62)	0.29	1.00(0.75-1.34)	0.99
**Ethnicity**						
White			Ref			
Black	1.84(1.17-2.89)	0.01	1.25(0.87-1.77)	0.23	0.82(0.58-1.15)	0.28
**Stroke subtype**						
Ischemic stroke						
Haemorrhagic stroke	1.00(0.56-1.80)	0.99	1.00(0.64-1.56)	0.99	0.74(0.48-1.14)	0.17
**Stroke severity†**						
Mild stroke			Ref			
Moderate to severe stroke	1.35(0.81-2.25)	0.25	1.65(1.12-2.43)	0.01	1.27(0.89-1.82)	0.19
**Physical disability‡**						
Mild disability						
Moderate to severe disability	1.41(0.86-2.32)	0.17	1.56(1.09-2.25)	0.02	1.33(0.94-1.88)	0.11
**Cognitive function§**						
Normal cognition			Ref			
Cognitive impairment	1.50(0.82-2.72)	0.19	1.38(0.88-2.16)	0.17	2.14(1.44-3.20)	<0.01
**Pre-stroke depression**						
No			Ref			
Yes	2.32(0.97-5.55)	0.06	1.92(0.95-3.88)	0.07	1.75(0.93-3.31)	0.08

Note: Multinomial logistic regression was used to identify factors associated with distinct trajectory groups. Group of no depression was the reference group.

*: Index of multiple deprivation, higher scores on the IMD indicate greater deprivation

†: Moderate to severe stroke: NIHSS score>4.

‡: Moderate to severe physical disability: Barthel Index<15.

§: Cognitive impairment: MMSE<24 or AMT<8.

Table S8 Associations between changes in patients' characteristics and courses of depressive symptoms up to 5-years after stroke (sensitivity analysis, exclude patients receiving antidepressant treatment)

	Incident depression		Recurrent depression		Persistent depression	
	**aOR(95%CI)**	**P value**	**aOR(95%CI)**	**P value**	**aOR(95%CI)**	**P value**
**Age**	0.99(0.98-1.01)	0.55	1.00(0.99-1.01)	0.97	0.98(0.97-0.99)	<0.01
**Socioeconomic status (IMD score)***	0.99(0.97-1.01)	0.20	1.02(1.00-1.04)	0.01	1.02(1.01-1.04)	<0.01
**Sex**						
Male			Ref			
Female	1.04(0.69-1.57)	0.86	1.09(0.80-1.50)	0.59	0.95(0.71-1.27)	0.72
**Ethnicity**						
White			Ref			
Black	1.80(1.14-2.84)	0.01	1.29(0.90-1.86)	0.17	0.80(0.57-1.14)	0.22
**Stroke subtype**						
Ischemic stroke						
Haemorrhagic stroke	1.02(0.57-1.83)	0.94	1.03(0.66-1.62)	0.89	0.77(0.50-1.19)	0.24
**Stroke severity†**						
Mild stroke			Ref			
Moderate and severe stroke	1.36(0.82-2.24)	0.23	1.57(1.07-2.30)	0.02	1.26(0.89-1.79)	0.20
**Change in physical disability‡**						
Mild at baseline and 3m			Ref			
Mild at baseline and moderate to severe at 3m	1.26(0.15-10.88)	0.83	10.77(4.15-27.94)	<0.01	4.64(1.64-13.14)	<0.01
Moderate to severe at baseline and mild at 3m	1.66(0.91-3.03)	0.10	1.70(1.08-2.69)	0.02	1.29(0.83-2.01)	0.27
Moderate to severe at baseline and 3m	2.57(1.12-5.92)	0.03	3.24(1.77-5.92)	<0.01	2.01(1.11-3.64)	0.02
**Change in cognitive function§**						
Normal at baseline and 3m			Ref			
Normal at baseline and impaired at 3m	2.22(0.89-5.58)	0.09	0.89(0.38-2.06)	0.78	1.79(0.85-3.80)	0.13
Impaired at baseline and normal at 3m	0.59(0.17-1.99)	0.39	1.43(0.77-2.64)	0.26	1.84(1.00-3.40)	0.05
Impaired at baseline and 3m	1.84(0.72-4.68)	0.20	1.26(0.59-2.71)	0.55	4.06(2.17-7.59)	<0.01
**Pre-stroke depression**						
No			Ref			
Yes	2.39(1.00-5.71)	0.05	2.16(1.07-4.39)	0.03	1.86(0;98-3.53)	0.06

Note: Multinomial logistic regression was used to identify factors associated with distinct trajectory groups. Group of no depression was the reference group.
*: Index of multiple deprivation, higher scores on the IMD indicate greater deprivation
†: Moderate to severe stroke: NIHSS score>4.
‡: Moderate to severe physical disability: Barthel Index<15.
§: Cognitive impairment: MMSE<24 or AMT<8.

Table S9 Associations between patient' baseline characteristics and courses of depression up to 5-years after stroke (sensitivity analysis, including patients survived more than 5 years)

	Transient depression		Recurrent depression		Persistent depression	
	aOR(95%CI)	P value	aOR(95%CI)	P value	aOR(95%CI)	P value
**Age**	1.00(0.98-1.02)	0.88	1.00(0.99-1.02)	0.46	0.98(0.97-0.99)	<0.01
**Socioeconomic status (IMD score)***	0.99(0.97-1.01)	0.42	1.03(1.01-1.04)	<0.01	1.02(1.01-1.04)	<0.01
**Sex**						
Male			Ref			
Female	0.97(0.64-1.47)	0.88	1.19(0.87-1.64)	0.27	1.08(0.80-1.44)	0.61
**Ethnicity**						
White			Ref			
Black	1.84(1.17-2.89)	0.01	1.14(0.80-1.62)	0.47	0.85(0.60-1.19)	0.33
**Stroke subtype**						
Ischemic stroke						
Haemorrhagic stroke	1.30(0.75-2.24)	0.35	1.06(0.68-1.65)	0.79	0.85(0.56-1.29)	0.44
**Stroke severity†**						
Mild stroke			Ref			
Moderate to severe stroke	1.22(0.74-2.02)	0.43	1.90(1.29-2.80)	<0.01	1.22(0.85-1.74)	0.28
**Physical disability‡**						
Mild disability						
Moderate to severe disability	1.36(0.82-2.26)	0.24	1.65(1.14-2.40)	<0.01	1.39(0.98-1.98)	0.06
**Cognitive function§**						
Normal cognition			Ref			
Cognitive impairment	1.87(1.04-3.36)	0.04	1.48(0.94-2.32)	0.09	2.12(1.40-3.19)	<0.01
**Pre-stroke depression**						
No			Ref			
Yes	2.27(0.96-5.38)	0.06	2.24(1.04-4.41)	0.04	3.03(1.73-5.33)	<0.01

Note: Multinomial logistic regression was used to identify factors associated with distinct trajectory groups. Group of no depression was the reference group.

*: Index of multiple deprivation, higher scores on the IMD indicate greater deprivation

†: Moderate to severe stroke: NIHSS score>4.

‡: Moderate to severe physical disability: Barthel Index<15.

§: Cognitive impairment: MMSE<24 or AMT<8.

Table S10 Associations between changes in patient' baseline characteristics and courses of depression up to 5-years after stroke (sensitivity analysis, included patients survived more than 5 years)

	Transient depression		Recurrent depression		Persistent depression	
	aOR(95%CI)	P value	aOR(95%CI)	P value	aOR(95%CI)	P value
**Age**	1.00(0.98-1.01)	0.69	1.00(0.99-1.01)	0.86	0.98(0.97-0.99)	<0.01
**Socioeconomic status (IMD score)***	0.99(0.97-1.01)	0.45	1.03(1.01-1.04)	<0.01	1.02(1.00-1.03)	0.01
**Sex**						
Male			Ref			
Female	0.94(0.62-1.44)	0.79	1.11(0.80-1.53)	0.53	1.05(0.78-1.40)	0.77
**Ethnicity**						
White			Ref			
Black	1.81(1.14-2.85)	0.01	1.17(0.81-1.68)	0.40	0.83(0.58-1.17)	0.28
**Stroke subtype**						
Ischemic stroke						
Haemorrhagic stroke	1.33(0.77-2.30)	0.31	1.13(0.72-1.77)	0.58	0.91(0.60-1.38)	0.66
**Stroke severity†**						
Mild stroke			Ref			
Moderate to severe stroke	1.20(0.74-1.97)	0.46	1.83(1.24-2.70)	<0.01	1.23(0.87-1.73)	0.25
**Change in physical disability‡**						
Mild at baseline and 3m			Ref			
Mild at baseline and moderate to severe at 3m	5.83(0.98-34.77)	0.05	20.68(6.23-68.66)	<0.01	9.60(2.72-33.85)	<0.01
Moderate to severe at baseline and mild at 3m	1.66(0.89-3.10)	0.11	1.70(1.05-2.73)	0.03	1.36(0.86-2.13)	0.19
Moderate to severe at baseline and 3m	3.79(1.61-8.94)	<0.01	4.82(2.56-9.08)	<0.01	2.74(1.45-5.18)	<0.01
**Change in cognitive function§**						
Normal at baseline and 3m			Ref			
Normal at baseline and impaired at 3m	1.35(0.47-3.88)	0.58	0.84(0.35-2.01)	0.69	1.58(0.71-3.48)	0.26
Impaired at baseline and normal at 3m	0.43(0.10-1.85)	0.26	1.81(0.98-3.36)	0.06	1.94(1.02-3.68)	0.04
Impaired at baseline and 3m	2.35(0.98-5.63)	0.06	1.28(0.58-2.84)	0.54	3.93(2.03-7.62)	<0.01
**Pre-stroke depression**						
No			Ref			
Yes	2.61(1.09-6.25)	0.03	2.39(1.20-4.77)	0.01	3.14(1.78-5.55)	<0.01

Note: Multinomial logistic regression was used to identify factors associated with distinct trajectory groups. Group of no depression was the reference group.
*: Index of multiple deprivation, higher scores on the IMD indicate greater deprivation
†: Moderate to severe stroke: NIHSS score>4.
‡: Moderate to severe physical disability: Barthel Index<15.
§: Cognitive impairment: MMSE<24 or AMT<8.

Table S11 Associations between patient' baseline characteristics and courses of depression up to 5-years after stroke (sensitivity analysis, including patients recruited before Mar 2014)

	Transient depression		Recurrent depression		Persistent depression	
	aOR(95%CI)	P value	aOR(95%CI)	P value	aOR(95%CI)	P value
**Age**	1.00(0.98-1.01)	0.86	1.00(0.99-1.01)	0.99	0.98(0.97-0.99)	<0.01
**Socioeconomic status (IMD score)***	0.99(0.97-1.01)	0.16	1.02(1.00-1.04)	0.01	1.02(1.01-1.03)	<0.01
**Sex**						
Male			Ref			
Female	1.04(0.69-1.57)	0.84	1.17(0.86-1.59)	0.31	1.18(0.89-1.57)	0.26
**Ethnicity**						
White			Ref			
Black	1.77(1.13-2.79)	0.01	1.32(0.93-1.87)	0.12	0.86(0.61-1.22)	0.40
**Stroke subtype**						
Ischemic stroke						
Haemorrhagic stroke	1.26(0.72-2.18)	0.42	0.97(0.63-1.52)	0.91	0.82(0.54-1.26)	0.37
**Stroke severity†**						
Mild stroke			Ref			
Moderate and severe stroke	1.00(0.61-1.65)	0.99	1.76(1.21-2.56)	<0.01	1.11(0.78-1.57)	0.57
**Physical disability‡**						
Mild disability			Ref			
Moderate to severe disability	1.37(0.84-2.23)	0.23	1.55(1.08-2.21)	0.02	1.25(0.89-1.76)	0.20
**Cognitive function§**						
Normal cognition			Ref			
Cognitive impairment	1.56(0.88-2.78)	0.13	1.34(0.87-2.06)	0.19	2.15(1.45-3.17)	<0.01
**Pre-stroke depression**						
No			Ref			
Yes	1.97(0.84-4.65)	0.12	1.52(0.76-3.03)	0.24	2.31(1.32-4.02)	<0.01

Note: Multinomial logistic regression was used to identify factors associated with distinct trajectory groups. Group of no depression was the reference group.

*: Index of multiple deprivation, higher scores on the IMD indicate greater deprivation.

†: Moderate to severe stroke: NIHSS score>4.

‡: Moderate to severe physical disability: Barthel Index<15.

§: Cognitive impairment: MMSE<24 or AMT<8.

Table S12 Associations between changes in patient' characteristics and courses of depression up to 5-years after stroke (sensitivity analysis, included patients recruited before Mar 2014)

	Transient depression		Recurrent depression		Persistent depression	
	**aOR(95%CI)**	**P value**	**aOR(95%CI)**	**P value**	**aOR(95%CI)**	**P value**
**Age**	1.00(0.98-1.01)	0.62	1.00(0.98-1.01)	0.51	0.98(0.97-0.99)	<0.01
**Socioeconomic status (IMD score)***	0.99(0.97-1.00)	0.13	1.02(1.00-1.03)	0.02	1.02(1.00-1.03)	0.01
**Sex**						
Male			Ref			
Female	1.00(0.66-1.51)	0.99	1.07(0.78-1.46)	0.66	1.12(0.84-1.50)	0.43
**Ethnicity**						
White			Ref			
Black	1.69(1.06-2.68)	0.03	1.38(0.97-1.98)	0.08	0.85(0.60-1.20)	0.36
**Stroke subtype**						
Ischemic stroke						
Haemorrhagic stroke	1.31(0.75-2.28)	0.34	1.02(0.65-1.59)	0.94	0.87(0.57-1.34)	0.54
**Stroke severity†**						
Mild stroke			Ref			
Moderate and severe stroke	0.99(0.61-1.62)	0.97	1.70(1.17-2.47)	<0.01	1.13(0.81-1.59)	0.48
**Change in physical disability‡**						
Mild at baseline and 3m						
Mild at baseline and worsened at 3m	2.48(0.49-12.62)	0.28	11.36(4.43-29.05)	<0.01	5.58(2.06-15.07)	<0.01
Moderate to severe at baseline and mild at 3m	1.58(0.86-2.89)	0.14	1.55(0.98-2.45)	0.06	1.14(0.73-1.80)	0.57
Moderate to severe at baseline and 3m	3.20(1.44-7.11)	<0.01	4.01(2.23-7.21)	<0.01	2.17(1.19-3.95)	0.01
**Change in cognitive function§**						
Normal at baseline and 3m			Ref			
Normal at baseline and impaired at 3m	2.32(0.96-5.62)	0.06	0.89(0.39-2.00)	0.77	1.66(0.78-3.54)	0.19
Impaired at baseline and normal at 3m	0.57(0.17-1.94)	0.37	1.54(0.86-2.75)	0.15	2.12(1.17-3.82)	0.01
Impaired at baseline and 3m	2.19(0.92-5.20)	0.08	0.96(0.44-2.09)	0.92	3.51(1.85-6.64)	<0.01
**Pre-stroke depression**						
No			Ref			
Yes	2.06(0.87-4.88)	0.10	1.63(0.81-3.29)	0.17	2.45(1.40-4.29)	<0.01

Note: Multinomial logistic regression was used to identify factors associated with distinct trajectory groups. Group of no depression was the reference group.
*: Index of multiple deprivation, higher scores on the IMD indicate greater deprivation.

†: Moderate to severe stroke: NIHSS score>4.
‡: Moderate to severe physical disability: Barthel Index<15.
§: Cognitive impairment: MMSE<24 or AMT<8.

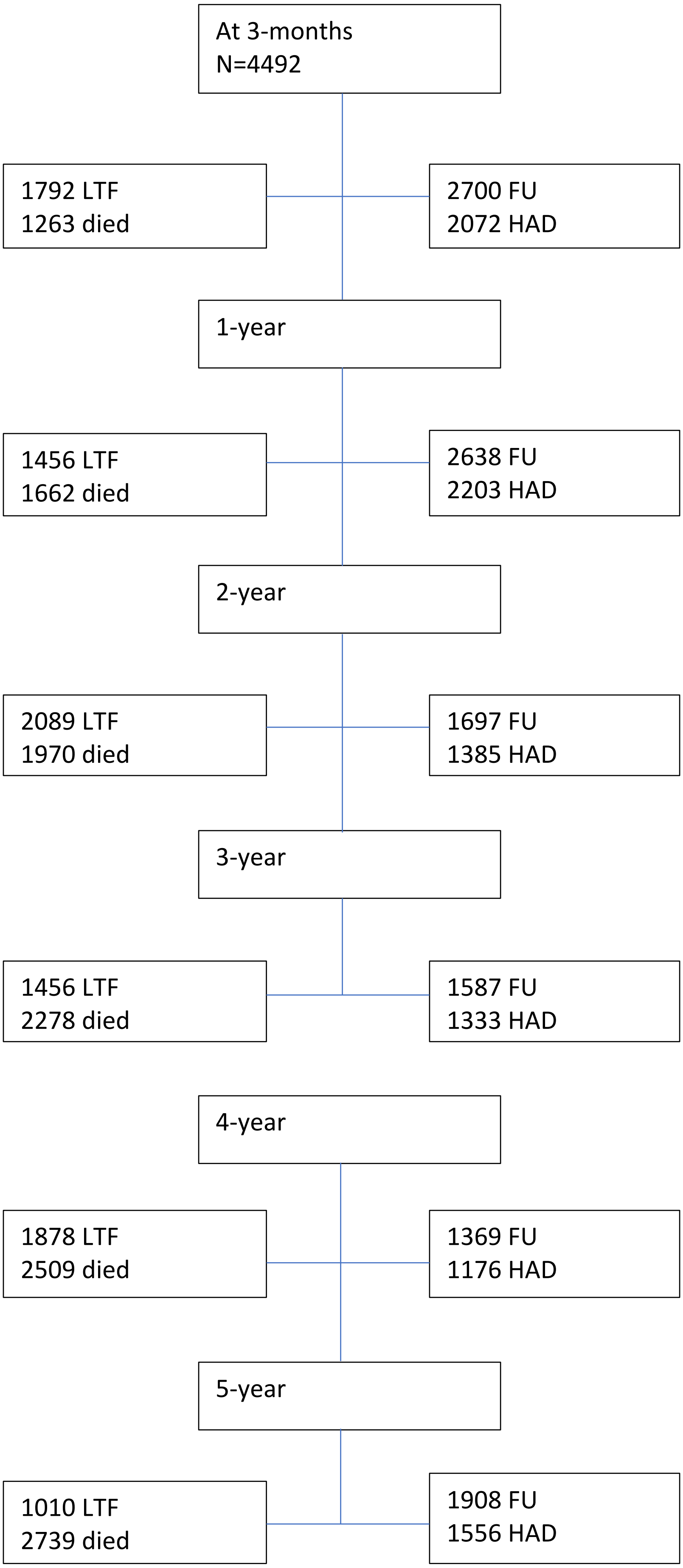
At 3-months
N=4492
1792 LTF
1263 died
2700 FU
2072 HAD
1-year
1456 LTF
1662 died
2638 FU
2203 HAD
2-year
2089 LTF
1970 died
1697 FU
1385 HAD
3-year
1456 LTF
2278 died
1587 FU
1333 HAD
4-year
1878 LTF
2509 died
1369 FU
1176 HAD
5-year
1010 LTF
2739 died
1908 FU
1556 HAD

Figure S1.Flow chart showing the number of stroke survivors with depression assessment at each time-point.

Note: N: number of patients registered; LTF: Lost to follow up; FU: completed the follow up; HAD: HAD completed.
